# Disease-Focused Research Using Stem Cells

**DOI:** 10.3390/biomedicines9111643

**Published:** 2021-11-08

**Authors:** Yohei Hayashi, Evgeniia Borisova

**Affiliations:** iPS Cell Advanced Characterization and Development Team, RIKEN BioResource Research Center, 3-1-1 Koyadai, Tsukuba 350-0074, Ibaraki, Japan; evgeniia.borisova@riken.jp

In this Special Issue of *Biomedicines* on disease-focused research using stem cells, we cover the latest conceptual and practical advances in stem cell-based therapies and disease modeling. Since the discovery of human embryonic stem cells in 1998, the furor of stem cell therapy or regenerative medicine has been long-lasting and undoubtedly intensifying with advances in gene editing, animal origin-free culture conditions, integration-free host cells transduction, and organoid technologies. The regenerative potentials of multipotent stem cells to reconstitute the entire cell system from progenitors up to mature cells are considered to be a promising source for damaged tissue substituting or in vitro disease modeling. The majority of multipotent stem cells circulating in the clinical industry are hematopoietic and mesenchymal stem cells. Various types of cells derived from human embryonic stem cell (hESC) and induced pluripotent stem cell (iPSC) are promising products for future cell therapies, due to their general capabilities of self-renewal and pluripotency [1].

Stem cell transplantation has never been out of sight ever since the first of its kind hematopoietic cell transplantations performed during post-Second World War. Hematopoietic stem cell transplantation (HSCT) [2] with the capacity of a life-long restoration of the entire hematopoietic system has pioneered the stem cell therapy field. Hematopoietic cell transplantation (HCT) became a therapy for patients with hematologic malignancies (chronic myeloid leukemia and acute lymphatic leukemia), non-malignant bone marrow disorders (aplastic anemia), and hemoglobinopathies (genetic diseases), as well as a cytotoxicity compensation for high-dose chemotherapy patients [3]. However, the risk of immune-mediated rejection by a host, also known as a graft-versus-host disease (GVHD), correlated with human leukocyte antigen (HLA)-disparity, and, additionally, the risk of cell cross-contamination and misidentification due to poor cell characterization, corrupts further research and clinical results. The cost-effective characterization of qualified stem cells is one of the major hurdles to expanding cell therapy platforms. In this Special Issue, Hong et al. presented a novel cost-effective microfluidic-based lab-on-chip system as an alternative to the common cost-intensive capillary gel electrophoresis method [4]. This lab-on-chip allows the in-house authentication of cell lines for generating robust and reproducible data. This study provides a proof of concept to build quality-controlled biobanks using hematopoietic stem cells as an explicit model.

The success of autologous and allogeneic HCT elevated the exploration of patients who might benefit from stem cell transplantations, as well as the exploration of potential stem cells to enter clinical development. Mesenchymal stem cells (MSCs), coresidents of the bone marrow niche together with hematopoietic stem cells, and the initial source suppliers of skin, bone, fat, and cartilage, are considered to be among the most promising adult stem cell types for cell or gene-based therapies. Due to the dual mechanism of action, immunomodulatory properties (induction of an anti-inflammatory environment) and the formation of differentiated cells at the site of injury [5], MSCs are the subject of serious research. The administration of MSCs has been approved for the treatment of GVHD in the European Union, Canada, and Australia. In addition to GVHD, an increasing number of studies and approximately a thousand MSC clinical trials ramify to most medical specialties, especially cardiovascular medicine, neurology, autoimmune diseases, and musculoskeletal diseases, highlighting that the application potential of MSCs is far from being exhausted [6]. Therefore, a review covering MSC therapy for nonischemic dilated cardiomyopathy (NIDCM) [7] and a research article developing MSCs as the next-generation cell-based therapeutics for atopic dermatitis (AD) [8] are included in this Special Issue.

Over 50% of all heart transplantations account for nonischemic dilated cardiomyopathy. The treatment for nonischemic dilated cardiomyopathy is generally limited to symptomatic therapeutic options due to its multifactorial etiology, leading to the eventual need for heart transplantation [9,10]. Although most clinical trials of MSC therapy on ischemic heart failure have focused on treating the direct myocardial damage with immune-mediated abnormalities underlying nonischemic dilated cardiomyopathy, the fittingness of MSCs to answer both of these issues remained poorly evaluated with scarce numbers of research. Hoeeg et al. presented a holistic review on efficacy and the mode of action of MSC therapy in NIDCM encompassing an up-to-date total of 27 studies (clinical and preclinical) with prospective questions and areas in need of further investigation [7].

On the other hand, based on the putative trophic and immunomodulatory mechanism of action, MSCs have been a strong therapeutics candidate for a broad spectrum of immunocompromised conditions. Limited data on the optimal therapeutic delivery of cell-based products warrants further research regarding MSCs’ in vivo mechanism of action. A novel approach proposed by Ryu B. et al. uses an MSC conditioned media concentrate as a therapeutic for atopic dermatitis. Secretions of hESC-derived multipotent MSCs were used to treat a mouse model of AD, showing the utility of hESC-derived multipotent MSCs as an immunoregulator having a significant relief effect in acute AD confirmed by protein expression and histological analyses. Being natural humoral immune regulators, MSC-based therapeutics may stand superior to conventional therapeutics, such as corticosteroids and antihistamines that are subject to various side effects [8].

The invention of iPSCs in 2006 [11] and the following development of pluripotent stem cell-derived products that are free of supply limitations was a breath of fresh air for regenerative medicine. Clear merits for using iPSCs in drug discovery and in cell-based regenerative medicine account for a stable track of large pharma activity and emerging clinical trials around reprogramming technology [12]. The iPSCs reprogramming approach may accurately recapitulate normal tissue-specific biology and disease models. Given the challenges of culture technology to meet manufacturing needs with a reproducible cell quality, we aim to identify and fill in the gaps in manufacturing requirements, cell types of characterization, and target indication [1]. Using differentiated disease-specific iPSCs in vitro and by comparing them to healthy donor iPSCs, we can identify responsible genes and screen for drug candidates [13,14,15,16]. The derivation of genome-engineered iPSC cell lines and according protocols are advancing iPSC technology further [17,18]. Genome editing studies performed as a disease-associated mutation correction in iPSCs or as a disease relevant mutation introduction in wild-type human iPSCs are steadily growing in numbers, addressing intractable and rare diseases, such as neurodegenerative disorders (Niemann-Pick type C, Parkinson’s disease, and Alzheimer’s disease), cystic fibrosis, skeletal dysplasia, and juvenile nephronophthisis. The invention of iPSCs brought new opportunities for tackling diseases that were previously compromised due to the restricted attainability of target tissues. Humanized patient-derived model systems linked with genome editing make room not only for applied, but also for basic research, expanding our knowledge about idiopathic and intractable disease pathogenesis.

The use of cells derived from iPSCs is particularly matching for degenerative diseases as the source for tissue grafts, because iPSCs can be established from essentially any person. Vanguard clinical trials of iPSC-derived transplants focusing on spinal cord injury and neovascular age-related macular degeneration validated the use of these cells clinically [12]. As for the novel disease indications and newly screened cellular regenerative products, Csobonyeiova et al. presented a thorough review about iPSC potencies towards osteoarthritis [19]. Osteoarthritis, being detrimental to the quality of life, faces unmet needs for the reconstruction of functional joint tissues. Owing to the diversity of cell populations within the joint structure, this clinical category holds a relative number of aspects to be yet optimized, including the choice of primary cell sources for chondrogenic differentiation and the establishment of suitable microenvironments, simultaneously becoming an area for extensive cross-disciplinary collaborations, such as tissue engineering for cartilage repair, bio-ink printing, bio-ceramic technology, organ-on-a-chip technology, and the development of specialized bioreactors.

Progress in generation methods and the gene manipulation of iPSCs prompted a comparison study of genetically modified human induced pluripotent stem cell-derived cardiomyocytes (hiPSC-CMs) and adipose-derived stromal cells (ADSCs) overexpressing antioxidative factors in a myocardial infarction model [20]. Cardiac function restoration after an ischemic heart injury predominantly occupies cell-based therapy clinical trials. Nevertheless, a decrease in the infarct size is hardly ever achieved. Therefore, Stępniewski et al. show a superior therapeutic potential of modified iPSCs expressing cardioprotective, pro-angiogenic, and immunomodulatory factors in the treatment of myocardial infarction.

Stem cell techniques and products could revolutionize heart, joint, and autoimmune disease patients’ care as shown in our *Biomedicines* Special Issue on “Disease-Focused Research Using Stem Cells”. Advances in stem cell technologies expand prospective target diseases to be tackled with pluripotent stem cells and offer novel modalities of treatment for patients with limited therapeutic options. Although the challenges to better describe and further enhance stem cell therapies efficacy by the use of more potent stem cell types, combined with gene modifying technologies and the optimization of manufacturing strategies, remain elusive, we anticipate many more benefits coming and partnerships within both industry and academia who are pushing for stem cell-based products development.

## Data Availability

Not applicable.
